# Proteomic analysis of *Artemisia annua* – towards elucidating the biosynthetic pathways of the antimalarial pro-drug artemisinin

**DOI:** 10.1186/s12870-015-0565-7

**Published:** 2015-07-09

**Authors:** Laura Bryant, Brian Flatley, Chhaya Patole, Geoffrey D. Brown, Rainer Cramer

**Affiliations:** Department of Chemistry, Whiteknights, Reading, RG6 6AD United Kingdom

**Keywords:** *Artemisia annua*, Artemisinin, Malaria, Mass spectrometry, Plant proteomics, Proteogenomics, Trichomes

## Abstract

**Background:**

MS-based proteomics was applied to the analysis of the medicinal plant *Artemisia annua*, exploiting a recently published contig sequence database (Graham *et al*. (2010) *Science* 327, 328–331) and other genomic and proteomic sequence databases for comparison. *A. annua* is the predominant natural source of artemisinin, the precursor for artemisinin-based combination therapies (ACTs), which are the WHO-recommended treatment for *P. falciparum* malaria.

**Results:**

The comparison of various databases containing *A. annua* sequences (NCBInr/*viridiplantae*, UniProt/*viridiplantae*, UniProt/*A. annua*, an *A. annua* trichome Trinity contig database, the above contig database and another *A. annua* EST database) revealed significant differences in respect of their suitability for proteomic analysis, showing that an organism-specific database that has undergone extensive curation, leading to longer contig sequences, can greatly increase the number of true positive protein identifications, while reducing the number of false positives. Compared to previously published data an order-of-magnitude more proteins have been identified from trichome-enriched *A. annua* samples, including proteins which are known to be involved in the biosynthesis of artemisinin, as well as other highly abundant proteins, which suggest additional enzymatic processes occurring within the trichomes that are important for the biosynthesis of artemisinin.

**Conclusions:**

The newly gained information allows for the possibility of an enzymatic pathway, utilizing peroxidases, for the less well understood final stages of artemisinin’s biosynthesis, as an alternative to the known non-enzymatic *in vitro* conversion of dihydroartemisinic acid to artemisinin. Data are available via ProteomeXchange with identifier PXD000703.

**Electronic supplementary material:**

The online version of this article (doi:10.1186/s12870-015-0565-7) contains supplementary material, which is available to authorized users.

## Background

There is growing interest in applying proteomics to organisms other than just those which are biomedically relevant and important species such as human, mouse or rat. However, one of the main hurdles for successful application of proteomics to an organism of interest is still the availability of a well annotated and curated (genomic) database that can be used to search the (mainly MS-based) proteomic data for protein identification in that organism. Thus, the field of proteogenomics is becoming increasingly important because of its ability to support the annotation of genomic sequence data by exploiting the information that is obtained through proteomics for the identification and characterization of the actual products of gene expression [1, 2].

Plant genomes can be highly complex and, in general, have been less well characterized than those from the animal kingdom, let alone those in the mammalian class, as mentioned above. For many plants, even those of high economic importance, the variability in the quality of available sequence databases can have a great effect on the power and depth of MS-based proteomic analysis. Consequently, it is desirable to understand and overcome the above limitations, leading to a more informative data set which can be constructed from the vast amount of data that is commonly obtained from large-scale MS-based proteomic analyses. Here, we have studied the organism *Artemisia annua*, which is a Chinese medicinal plant endemic to the Northern parts of China. *A. annua* is crucial to world health programs as it is currently the sole source for biosynthetically produced artemisinin, the antimalarial pro-drug that has now been the last line of defence against malaria for several decades.

The sesquiterpene lactone, artemisinin, is the precursor for artemisinin-based combination therapies (ACTs), which are the WHO-recommended treatment for *P. falciparum* malaria [3]. Due to its unique mode of action, artemisinin has been found to be effective against the asexual (blood) stage of the malarial parasite’s life cycle [4], which has acquired resistance to the older generation of antimalarial drugs. Between 2005 and 2013, the number of ACT treatments procured by the public and private sectors in endemic countries rose 36-fold, reaching a total of 392 million in 2013 [3].

Thus, the reliable supply of artemisinin as the precursor compound for the active ingredient of ACTs is of crucial importance in the fight against malaria. However, the current production of artemisinin is compromised by the fact that it is reliant on cultivation of *A. annua*. Thus, in areas where *A. annua* is competing against food for use of the land, the rise in food prices will cause farmers/extractors to have less of an incentive to grow *A. annua*. Furthermore, farmers/extractors need to decide whether or not to plant *A. annua* some 14 months before the drug can be produced. Finally, floods such as those frequently experienced in China and Vietnam make the supply of ACTs unpredictable [5]. Taking these and other factors into account it is highly desirable to develop a method of production of artemisinin that is not dependent on *A. annua* cultivation, can be scaled up at short notice and is inexpensive.

In order to achieve these goals, two different approaches (as well as a hybrid of both) are now described. One of these approaches is based on chemical synthesis of artemisinin from commercially available starting materials such as the monocyclic monoterpene, isopulegol. However, artemisinin production solely by such chemical means is complex and expensive, and therefore, has so far not supplanted the agricultural production of artemisinin as the favoured production method. An alternative to chemical synthesis is the employment of genetically engineered fast-growing organisms that produce artemisinin in high quantities. In this approach, the biosynthetic pathway to artemisinin needs to be expressed in an organism such as yeast by genetic modification of the host with the relevant genes from this pathway. There are several aspects of this bio-engineering approach that are important for its success, including the viability and fast growth of the engineered organism as well as the compartmentalization/secretion of the biosynthetic product in such a way that harvesting becomes an easy enterprise, to name but a few. For all this, a comprehensive knowledge of the biosynthetic pathway to artemisinin in *A. annua* is paramount, but unfortunately this goal has not yet been achieved (in particular, the final steps of the biosynthesis are still not completely defined). However, very significant advances have been achieved in spite of this, as demonstrated by two recent publications describing the bio-engineering of artemisinin-producing tobacco [6] and that of artemisinic acid-producing yeast [7]. While the former concludes that current yields in tobacco are still significantly below the percentage dry weight levels obtainable from *A. annua* (by a factor of more than 1000), the latter reports an incomplete biosynthesis to artemisinic acid, which then needs to be chemically converted to artemisinin. Although great progress has been made recently using the latter approach [8], it remains the case that expensive and complex chemical synthetic steps are still needed in the final stages of producing artemisinin.

Crop-breeding programs, on the other hand, have produced new varieties of *A. annua*, such as Artemis, with a consistently high yield of artemisinin (1 %); and on-going projects are aiming to push this yield even higher. However, it is quite clear that both the crop-breeding and fermentation/chemical synthesis strategies would benefit from full knowledge of the biosynthetic route to artemisinin.

The biosynthesis of artemisinin is thought to be localized within the glandular trichomes of the plant which are found on the leaf surface [9]. Trichomes are leaf hairs which originate from the protrusion of specialized epidermal cells on various parts of the plant, including leaves and stems. Typically, trichomes are divided into 2 categories: non-glandular and glandular. Non-glandular trichomes are involved in processes such as water absorption and seed dispersal, whereas glandular trichomes are involved in processes connected with secondary metabolites including biosynthesis, storage and secretion [10, 11]. There is currently much interest in taking advantage of the glandular trichomes’ biosynthetic function in order to produce compounds, which have pharmaceutical use, such as artemisinin.

The glandular secretory trichome of *A. annua* is a 10-celled biseriate which consists of two basal cells, two stalk cells and three pairs of secretory cells. It is within this multi-cellular glandular trichome that biosynthesis of artemisinin occurs [12]. By comparing the amount of artemisinin extracted from glanded and glandless biotypes of *A. annua*, Duke *et al.* revealed that all extractable artemisinin was localized in the subcuticular space of the captitate glands [9]. After extraction, no artemisinin was found in the glandless biotype, thereby implying that the biosynthesis of artemisinin is localized in the glandular secretory trichomes [9].

The current study focuses on the analysis of the trichome proteome of *A. annua*, exploiting the recently published EST and unigene sequence databases published by Graham *et al*. [13]. This dataset is evaluated in comparison to four other databases (three protein sequence databases and one trichome-specific Trinity contig database [14]) with regard to their suitability for proteomic analysis. In addition, a comparison is made with the only other MS-based proteomic study of *A. annua* trichomes, which used another different EST data set assembly [10]. More importantly, the current study provides a significantly extended proteomic data set for *A. annua*, potentially leading to a better understanding of the trichome machinery and its role in the production of artemisinin.

## Results

### Isolation of glandular trichomes

The protocol that was applied for the isolation of glandular trichomes is based on the process of glass bead abrasion which has been described previously [10, 15, 16]. In these earlier studies, it was reported that the majority of the cell material enriched by this method represented glandular trichomes albeit with a significant remainder of non-glandular trichomes [15, 16]. Our simplified method, omitting the sucrose gradient fractionation, confirmed these findings as shown by environmental scanning electron microscopy (ESEM) analysis (see figure in Additional file 1), indicating that predominantly glandular trichome material had been obtained from the treated leaves. However, ESEM also revealed a significant number of glandular trichomes left on the leaf material after glass bead abrasion (Additional file 1). It is assumed therefore that there are only small differences between the ‘trichome-depleted’ and 'whole leaf' samples. Consequently, the data presented and discussed here are mainly based on the analysis of the ‘trichome-enriched’ material and its comparison solely with the ‘trichome-depleted’ sample material, as the technical replicate analysis of these samples showed a similarly low relative standard deviation of around 2.5 % for the number of identified proteins. By contrast, this number was around 8 % for the 'whole leaf' replicate samples (see table in Additional file 2).

### LC-MS/MS analysis

The triplicate LC-MS/MS runs showed that for all triplicates, the majority of protein identifications (using the York Artemis contig database) were also obtained in the other two respective replicates, indicating an acceptable level of technical reproducibility. For the trichome-enriched sample analysis of the merged triplicate LC-MS/MS data a total of 671 contig ‘protein families’ entries (see Additional file 3) were significantly matched while for the trichome-depleted and whole leaf sample analysis this number was slightly higher at 774 and 749, respectively.

The greatest number of protein identifications was obtained by searching the York Artemis contig database and the *A. annua* trichome Trinity contig database. However, as there is no functional annotation provided in these databases, the data was also searched against the UniProtKB database, restricted to *viridiplantae*. These searches led to a total of 319 protein (family) identifications for the trichome-enriched samples (see Additional file 4) while for the trichome-depleted and whole leaf sample analysis this number was again slightly higher at 417 and 408, respectively.

Decoy database searches using the default option in Mascot (reversed protein sequences) showed that the false discovery rate (FDR) for peptide matches above identity threshold was between 1.5 % and 1.9 % for all Artemis contig decoy database searches while the FDR for the UniProtKB (taxonomy: *viridiplantae*) decoy database search of the trichome-enriched sample data was ~9 % (see Table 1). Interestingly, the FDR for the corresponding Trinity contig database search was 3.2 % (see Table 1).

**Table 1:** Protein identification search results from the MS/MS data of trichome-enriched *A. annua* sample material

Target database	# of protein family hits	# of peptides matched above identity threshold in target database	# of peptides matched above identity threshold in decoy database	FDR
York Artemis contigs (116,303 sequences)	671	3072	58	1.9 %
Artemisia trichome Trinity contigs (150,282 sequences)	684	2636	85	3.2 %
NCBInr *viridiplantae* (1,079,491 sequences)	419	1286	60	4.7 %
UniProt *A. annua* (118 sequences)	17	179	11	6.2 %
UniProt *viridiplantae* (32,329 sequences)	319	1271	112	8.8 %

### The trichome-enriched proteome of Artemisia annua

**Fig. 1:**
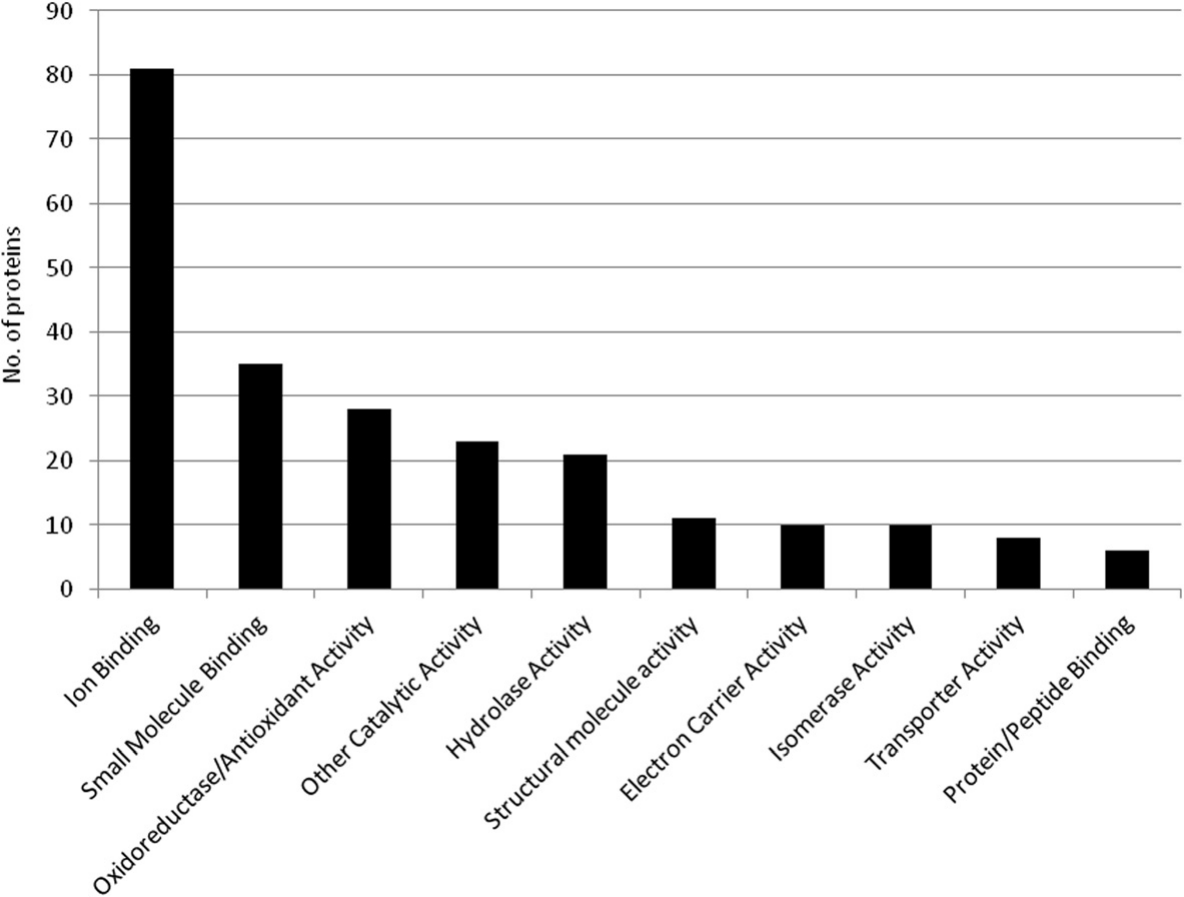
Functional classification (GO terms) of the Masoct-identified proteins from the trichome-enriched sample material, using the UniProtKB database (taxonomoy: *viridiplantae*). Mascot search results were submitted to Percolator with an ‘expect cut-off’ threshold of 0.05

In general, the vast majority of proteins identified previously from *A. annua* by Wu *et al.* were also found in our datasets [10]. Notably, in the trichome-enriched sample data, we found amongst others a similarly large number of ATPases/ATP synthases and oxidoreductases (*e.g.* four ferredoxins), as well as many proteins involved in translation and transcription and also in proteolysis and the proteosome. Furthermore, several kinases and phosphatases were also identified. Figure 1 displays a rough molecular functional classification (GO terms) of the UniProtKB (taxonomoy: *viridiplantae*)-identified proteins from the trichome-enriched sample material after submission to Percolator and setting the ‘expect cut-off’ threshold to 0.05.

Importantly, several known and putative enzymes on the biosynthetic pathway to artemisinin have been found when searching the UniProtKB database entries restricted to the taxonomy *A. annua*. These include: peroxidase 1 (UniProt # Q84UA9), artemisinic aldehyde delta-11(13) reductase (DBR2; UniProt # C5H429), amorpha-4,11-diene synthase (ADS; UniProt # Q9AR04), 2-alkenal reductase (UniProt # C0LNV1), HMG-CoA reductase (HMGR; UniProt # Q9SWQ3), and putative heme-binding cytochrome P450 (UniProt # Q2EPZ0), as listed in Table 2. Interestingly, most of these enzymes were not identified by searching the entire clade *viridiplantae* of the UniProtKB database. However, most of them and several other proteins related to the biosynthetic pathway to artemisinin were found searching the data against the contig database and by using the BLASTx search utility for functional annotation.

**Table 2:** Significant protein matches obtained from searching MS/MS data of the trichome-enriched *A. annua* sample material (against UniProtKB database; taxonomy: *A. annua*)

Accession #	Score	Mass	Description
Q84UA9	1811	35892	Peroxidase 1
Q4Q028	1037	25593	Ribulose bisphosphate carboxylase large chain (Fragment)
A5HSG6	934	26996	Chloroplast light-harvesting chlorophyll a/b-binding protein (Fragment)
B2CNV6	393	28433	Actin 1 (Fragment)
A0A3A2	273	10794	Ribulose bisphosphate carboxylase small chain (Fragment)
A5HSG5	219	11402	Histone H4
C5H429	177	42705	Artemisinic aldehyde delta-11(13) reductase
O22036	84	16124	1-aminocyclopropane-1-carboxylate oxidase (Fragment)
D2X5S7	76	10971	Putative ubiquitin-like protein
G0WKM6	54	26819	Allene oxide cyclase
Q9AR04	45	64406	Amorpha-4,11-diene synthase
D2X5N3	45	21350	Glyceraldehyde-3-phosphate dehydrogenase (Fragment)
C0LNV1	43	38761	2-alkenal reductase
A5HSG7	33	14862	Putative ubiquitin-conjugating enzyme
A0A3A1	24	24364	Chitinase-like protein (Fragment)
Q9SWQ3	13	62512	HMG-CoA reductase
Q2EPZ0	13	61373	Putative heme-binding cytochrome P450

Although this study was first and foremost designed to investigate the usefulness of the York Artemisia contig database and other databases for proteomic analyses, a preliminary comparison between the protein abundances of the trichome-enriched and trichome-depleted sample material was also thought to be useful for both (i) restricting the number of BLAST searches; and (ii) providing some means of focusing on trichome- and thus potentially pathway-specific enzymes for further studies. In order to compare the protein abundances between the trichome-enriched and trichome-depleted sample material the contigs’ emPAI values were determined. For their calculated quotients, Tables 3 and 4 present the 20 proteins that gave the highest and lowest values, respectively, while Tables 5 and 6 present the 10 proteins with the highest emPAI values from the trichome-enriched sample material that were not detected in the trichome-depleted sample material and *vice versa*.

**Table 3:** Highly abundant proteins of the trichome-enriched compared to the trichome-depleted sample material

Gi number	EmPAI quotient^a^	Access. number (no taxonomy)	Protein name (no taxonomy)	Access. number (Tax.: Artemisia)	Protein name (Tax.: Artemisia)
259726479	6.42	ACZ51443.1	Peroxidase protein [Mikania micrantha]	AAO45182.1	Peroxidase 1 [Artemisia annua]
259544254	5.70	XP_002285593.1	Aspartic proteinase nepenthesin-2 [Vitis vinifera]		
259560512	4.79	Q9XIV8.1	Peroxidase N1	AAO45182.1	Peroxidase 1 [Artemisia annua]
259867266	3.86	XP_004135957.1	Photosystem II stability/assembly factor HCF136, chloroplastic-like [Cucumis sativus]		
259537187	3.76	XP_004251245.1	Elongation factor 1-beta 1-like [Solanum lycopersicum]		
259859266	3.73	XP_003538726.1	Thylakoid lumenal protein At1g12250, chloroplastic-like [Glycine max]		
259714573	3.08	XP_002523024.1	Amino acid binding protein, putative [Ricinus communis]		
259551155	2.97	CAH17986.1	Peroxidase POA1 [Capsicum annuum]	AAO45182.1	Peroxidase 1 [Artemisia annua]
259554365	2.59	ABK92641.1	Unknown [Populus trichocarpa]		
259530226	2.35	O04287.1	Peptidyl-prolyl cis-trans isomerase FKBP12		
259724484	2.13	CAA81073.1	H-protein [Flaveria cronquistii]		
259543684	2.06	ABV26711.1	Cyclophilin [Gerbera hybrid cultivar]		
259527950	1.89	AAD11576.1	Lectin 3 [Helianthus tuberosus]	AFU82531.1	CjMDR1 protein, partial [Artemisia tridentata]
259557766	1.81	XP_006350796.1	Thylakoid lumenal 15 kDa protein 1, chloroplastic-like [Solanum tuberosum]		
259561193	1.81	AEF01110.1	Cyclophilin 2 [Tagetes patula]		
259539505	1.73	XP_002301776.1	Predicted protein [Populus trichocarpa]		
259534802	1.66	XP_004249062.1	Macrophage migration inhibitory factor homolog isoform 2 [Solanum lycopersicum]		
259531358	1.64	EOY05690.1	Cysteine synthase [Theobroma cacao]		
259732623	1.62	XP_003544567.1	Fructose-bisphosphate aldolase, cytoplasmic isozyme 1-like [Glycine max]		
259544645	1.58	BAD10859.1	Cysteine protease [Aster tripolium]		

Please note: BLASTx searches were used to find protein homologues in other species without taxonomy restriction (columns 3 & 4) and with taxonomy restriction to Artemisia (columns 5 & 6). ^a^This is the quotient of the emPAI values of the merged trichome-enriched samples divided by the emPAI values of the merged trichome-depleted samples. Only contigs identified in all triplicates were considered.

**Table 4:** Highly abundant proteins of the trichome-depleted compared to the trichome-enriched sample material

Gi number	EmPAI quotient^a^	Acc. number (no taxonomy)	Protein name (no taxonomy)	Acc. number (Tax.: Artemisia)	Protein name (Tax.: Artemisia)
259558480	0.14	CAE82295.1	Catalase [Homogyne alpina]		
259720159	0.20	AFK36692.1	Unknown [Medicago truncatula]		
259732783	0.27	XP_004140097.1	Ribosome-recycling factor, chloroplastic-like [Cucumis sativus]		
259539861	0.33	ADG96009.1	Beta-1,3-glucanase PR2 [Chrysanthemum x morifolium]		
259732073	0.34	YP_007353779.1	Component of cytochrome b6/f complex (chloroplast) [Chrysanthemum x morifolium]	YP_007624808.1	Cytochrome f (chloroplast) [Artemisia frigida]
259542667	0.35	P29790.1	ATP synthase gamma chain, chloroplastic		
259550993	0.37	XP_003547929.1	Ferredoxin--NADP reductase, leaf isozyme, chloroplastic-like isoform 1 [Glycine max]	AFO64618.1	Cytochrome P450 reductase [Artemisia annua]
259863294	0.38	NP_193769.1	Putative elongation factor Tu [Arabidopsis thaliana]		
259530332	0.40	P00290.1	Plastocyanin		
259532611	0.42	XP_002271791.1	Photosystem II 10 kDa polypeptide, chloroplastic [Vitis vinifera]		
259544910	0.43	ABC41657.1	Carbonic anhydrase 2 [Flaveria pringlei]		
259533513	0.43	AAO42615.1	Ferredoxin [Helianthus annuus]		
259535874	0.43	XP_002299232.1	Predicted protein [Populus trichocarpa]		
259553333	0.44	XP_004228903.1	Photosystem II CP47 chlorophyll apoprotein-like [Solanum lycopersicum]	YP_007624821.1	Photosystem II P680 chlorophyll A apoprotein (chloroplast) [Artemisia frigida]
259529761	0.44	BAL14665.1	Elongation factor 1-alpha [Chrysanthemum seticuspe f. boreale]	ACP28182.1	Elongation factor 1-alpha [Artemisia annua]
259529664	0.49	ACO35888.1	Ribulose-1,5-bisphosphate carboxylase/oxygenase small subunit [Ageratina adenophora]	ABJ74187.1	Chloroplast ribulose-1,5-bisphosphate carboxylase/oxygenase small subunit [Artemisia annua]
259734867	0.49	CBI19198.3	Unnamed protein product [Vitis vinifera]		
259531744	0.49	P46486.1	Photosystem I reaction center subunit III, chloroplastic		
259542148	0.53	BAA04633.1	PSI-H precursor [Nicotiana sylvestris]		
259563039	0.54	ABW80752.1	Chloroplast ribulose 1,5-bisphosphate carboxylase/oxygenase activase [Flaveria bidentis]		

Please note: BLASTx searches were used to find protein homologues in other species without taxonomy restriction (columns 3 & 4) and with taxonomy restriction to Artemisia (columns 5 & 6). ^a^This is the quotient of the emPAI values of the merged trichome-enriched samples divided by the emPAI values of the merged trichome-depleted samples. Only contigs identified in all triplicates were considered.

**Table 5:** Ten highest abundant proteins detected in the trichome-enriched samples only^a^

Gi number	Accesssion number (no taxonomy)	Protein name (no taxonomy)	Accession number (Tax.: Artemisia)	Protein name (Tax.: Artemisia)
259723715	YP_007624803.1	Ribulose-1,5-bisphosphate carboxylase/oxygenase large subunit (chloroplast) [Artemisia frigida]	YP_007624803.1	Ribulose-1,5-bisphosphate carboxylase/oxygenase large subunit (chloroplast) [Artemisia frigida]
259717416	XP_004240055.1	Cationic peroxidase 1-like [Solanum lycopersicum]	AAO45182.1	Peroxidase 1 [Artemisia annua]
259711439	AAM27915.1	Carbohydrate oxidase [Helianthus annuus]		
259552602	ACZ74626.1	Beta-1,3-glucanase form RRII Gln 3 [Hevea brasiliensis]		
259552323	XP_002514900.1	Nuclear transport factor, putative [Ricinus communis]		
259732733	XP_004302854.1	Chlorophyll a-b binding protein 40, chloroplastic-like [Fragaria vesca subsp. vesca]	ABQ32304.1	Chloroplast light-harvesting chlorophyll a/b-binding protein [Artemisia annua]
259737301	XP_002276749.1	20 kDa chaperonin, chloroplastic [Vitis vinifera]		
259558851	XP_004159350.1	ruBisCO large subunit-binding protein subunit beta, chloroplastic-like [Cucumis sativus]		
259717225	XP_002283307.1	psbP domain-containing protein 6, chloroplastic [Vitis vinifera]		
259728302	XP_002272847.1	Peroxidase N1 [Vitis vinifera]	AAO45182.1	Peroxidase 1 [Artemisia annua]

Please note: BLASTx searches were used to find protein homologues in other species without taxonomy restriction (columns 2 & 3) and with taxonomy restriction to Artemisia (columns 4 & 5). ^a^These proteins have been detected in all three trichome-enriched but none of the trichome-depleted samples.

**Table 6:** Ten highest abundant proteins detected in the trichome-depleted samples only^a^

Gi number	Accession number (no taxonomy)	Protein name (no taxonomy)	Accession number (Tax.: Artemisia)	Protein name (Tax.: Artemisia)
259550200	EOY18549.1	ATP synthase D chain, mitochondrial [Theobroma cacao]		
259729601	XP_002965312.1	Hypothetical protein SELMODRAFT_439163 [Selaginella moellendorffii]	AFU82552.1	Heat shock protein, partial [Artemisia tridentata]
259540721	AAC84136.1	Ribosomal protein L12 [Cichorium intybus]		
259545610	XP_003543611.1	60S ribosomal protein L28-2 [Glycine max]		
259719587	AET22433.1	Glutamate synthase [Camellia sinensis]		
259870147	XP_004503350.1	40S ribosomal protein S2-3-like [Cicer arietinum]	ABQ32304.1	Chloroplast light-harvesting chlorophyll a/b-binding protein [Artemisia annua]
259543403	CAA65042.1	Chlorophyll a/b-binding protein CP26 in PS II [Brassica juncea]	ABQ32304.1	Chloroplast light-harvesting chlorophyll a/b-binding protein [Artemisia annua]
259736780	XP_003547929.1	Ferredoxin--NADP reductase, leaf isozyme, chloroplastic-like isoform 1 [Glycine max]	AFO64618.1	Cytochrome P450 reductase [Artemisia annua]
259532505	AEC10962.1	40S ribosomal protein s10 [Camellia sinensis]		
259538077	XP_002301623.1	Predicted protein [Populus trichocarpa]		

Please note: BLASTx searches were used to find protein homologues in other species without taxonomy restriction (columns 2 & 3) and with taxonomy restriction to Artemisia (columns 4 & 5). ^a^These proteins have been detected in all three trichome-depleted but none of the trichome-enriched samples.

**Fig. 2:**
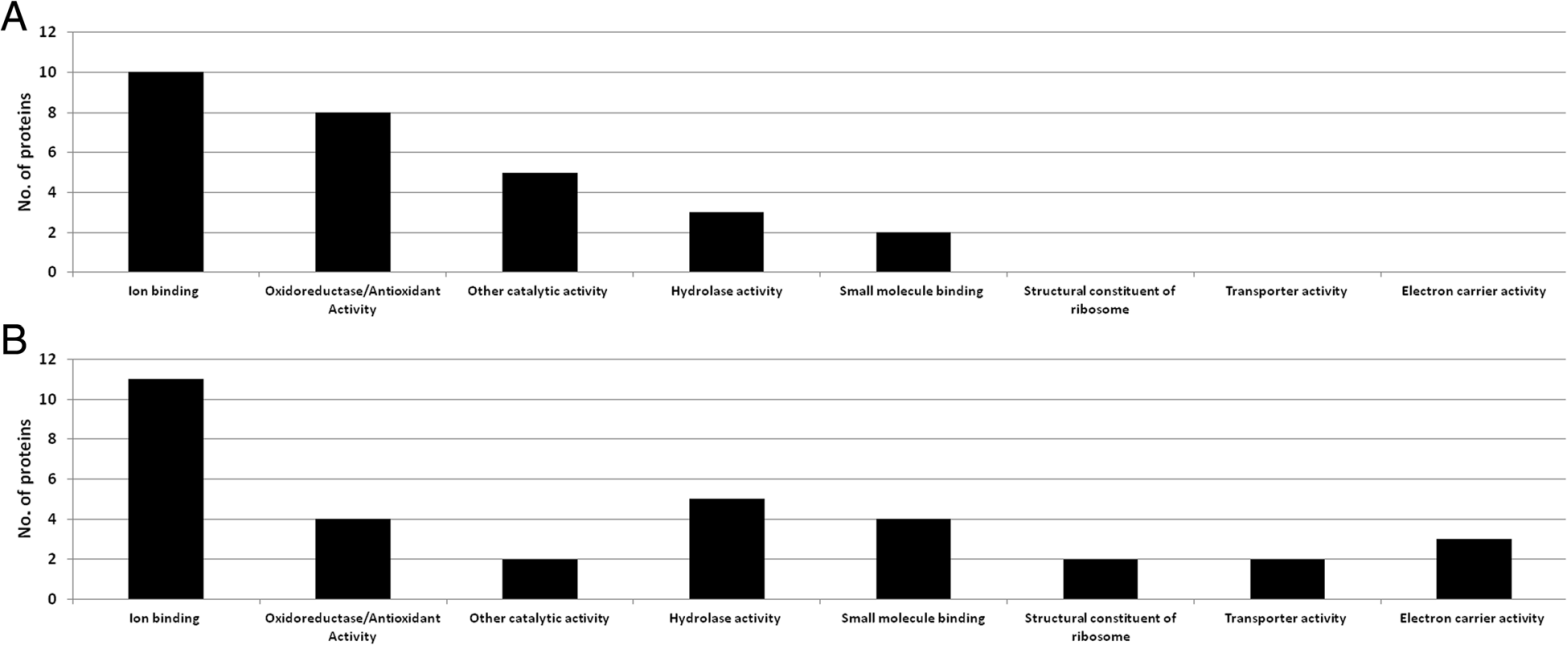
Functional classification (GO terms) of the Mascot-identified proteins from the trichome-enriched and trichome-depleted sample material, using the Artemis contig database: **a** for protein identifications of Tables 3 and 4 with higher abundance in trichome-enriched sample material; **b** for protein identifications of Tables 5 and 6 with lower abundance in trichome-enriched sample material. Mascot search results were submitted to Percolator with an ‘expect cut-off’ threshold of 0.05 and filtered using a minimum number of significant sequences of 2

Figure 2 displays the functional classification of the proteins in Tables 3, 4, 5, 6 according to their abundance levels (Fig. 2a: higher abundance in trichome-enriched sample material; Fig. 2b: lower abundance in trichome-enriched sample material).

## Discussion

The number of protein families identified in this study from trichome-enriched samples represents an increase by a factor of 7–8 when compared to the protein identification data achieved by the only other previously published larger proteomics study with *A. annua* by Wu *et al.* [10]. The increase is even higher (12-14-fold) if these results are compared to the EST search results of “non-redundant” protein hits in the earlier study [10]. This order-of-magnitude increase in proteome coverage is arguably due to the different technical approaches adopted (nanoUHPLC-ESI MS/MS *vs.* gel-based MALDI MS/MS) and also to the databases which have been employed. For instance, the study by Wu *et al.* was restricted to the separation of proteins by 2DE using a pH gradient of 4–7 [10]. For the databases used in the present study, the translated York contig database searched by Mascot comprises 85,508,608 residues, which equates to an average of ~123 residues per translated contig sequence, while Wu *et al.* used an in-house EST database, resulting in 49,389,486 residues and 2,060,880 sequences, *i.e.* an average of ~24 (more than 5-fold less) residues per translated EST sequence. The greater fragmentation (and larger number of sequences) of the latter database negatively affects protein identification, which is partially reflected in the different individual ion scores that were necessary for identity or extensive homology (p < 0.05) in the two studies. For the present study, these had to be only >30, while in the study by Wu *et al.* this threshold was reported to be >41 [10].

The results obtained from the MS/MS data searches using different databases (*cf*. Table 1) demonstrate the importance of the availability and quality of organism-specific (genomic) sequence data for proteomic analysis. When the NCBInr database was searched with the taxonomy restriction of *viridiplantae*, *i.e.* similarly to the work of Wu *et al.* [10], 419 protein family matches were obtained with an FDR of 4.7 %. Searching a custom database restricted to the UniProtKB entries for *A. annua* (created on 19. January 2012; 118 sequences, 41,707 residues) resulted in 17 protein family hits for the trichome-enriched sample data with 11 peptide matches above identity threshold for the decoy database search, *i.e.* an FDR of 6.2 %. The highest FDR (~9 %) was obtained from the UniProtKB (taxonomy: *viridiplantae*) decoy database search.

Interestingly, the Trinity trichome contig database search of the MS/MS data of the trichome-enriched sample material (see Additional file 5) yielded a slightly higher number of protein family hits compared to the York Artemis contig database search (Table 1) but had far less peptides matched above the identity threshold and a substantially higher FDR (3.2 %) as well as 27 more peptides matched above the identity threshold in the decoy database search, potentially negating the slightly higher number of protein family hits. Thus, the York Artemis contig database appears to be the best sequence database for proteomic analyses.

Overall, the above analysis shows that using large common protein sequence databases, even if well curated and/or for proteomic analysis restricted to a specific organism or clade, can easily result in high false discovery rates for organisms that have been less well sequenced and characterized. They also show that high quality (genomic) sequence information for these organisms provides a significant advantage if one wants to achieve greater proteome coverage and lower numbers of spurious protein identifications.

**Fig. 3:**
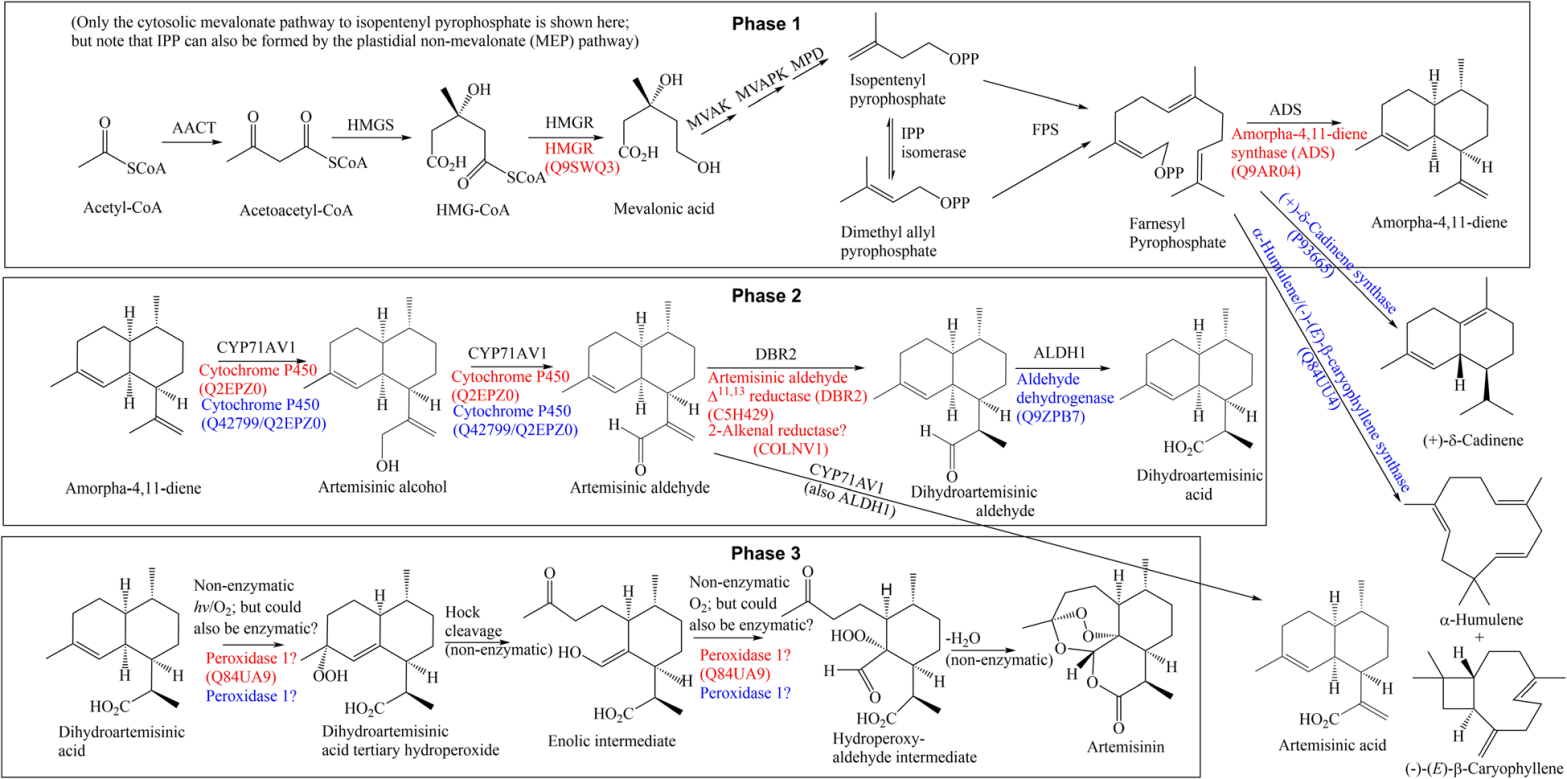
The biosynthesis of artemisinin, as it is currently best understood, depicted in three phases. Enzymes in red were identified through Mascot searches of the MS data using the taxonomy *A. annua* while enzymes in blue were identified using the taxonomy *viridiplantae*. For the latter, if needed, additional homology searching was applied, using BLASTp with ‘Artemisia’ as organism (E value < 10^−47^). AACT – acetoacetyl-CoA thiolase; HMGS – (*S*)-3-hydroxy-3-methylglutaryl-CoA synthase; HMGR – (*S*)-3-hydroxy-3-methylglutaryl-CoA reductase; MVAK – mevalonate kinase; MVAPK – mevalonate-5-phosphate kinase; MPD - mevalonate-5-pyrophosphate decarboxylase; FPS – farnesyl pyrophosphate synthase; ADS – amorpha-4,11-diene synthase; CYP71AV1 - amorpha-4,11-diene 12-hydroxylase; DBR2 – artemisinic aldehyde Δ^11,13^ reductase; ALDH1 – aldehyde dehydrogenase 1

The protein abundance analysis between the trichome-enriched and -depleted samples using their emPAI values shows that peroxidases have far greater abundance within the trichome-enriched sample material. This data could be relevant for the elucidation of the final (oxidative) step in the biosynthesis of artemisinin (see Phase 3 in Fig. 3), which is thought to proceed most likely *via* the precursor of dihydroartemisinic acid and its derived tertiary allylic hydroperoxide. It has been found that all the reactions depicted in the final phase of the biosynthesis of artemisinin in Fig. 3 can proceed non-enzymatically *in vitro*, and it has been suggested that this might also be the case *in vivo*. However, the over-expression of peroxidases arguably indicates the involvement of enzymes in this final step in the biosynthesis of artemisinin.

In addition, cyclophilins which usually catalyse the isomerisation of peptidic bonds from the *trans* to the *cis* form at proline residues were found to be in greater abundance in the trichome-enriched sample material.

In general, there seemed to be a relatively higher level of ribosomal proteins, ATP/glutamate synthases, and proteins with transporter and electron carrier activity in the trichome-depleted sample material (see Fig. 2). This is probably partly due to the dominance of the above-described proteins in the trichome-enriched sample material, which catalyse trichome-specific biosynthetic and metabolic processes. As can be seen in Fig. 2 for the top 10/20 most abundant proteins, there is a far greater number in the categories ‘oxidoreductase/antioxidant activity’ and ‘other catalytic activity’ for the trichome-enriched sample material than for the trichome-depleted sample material.

Finally, in both trichome-enriched and trichome-depleted samples there was a significant background of chloroplastic proteins associated with photosynthetic processes, which conforms with a previous *A. annua* transcriptomics study, where a large number of transcripts matching photosynthetic homologues were indentified [17]. There were comparatively more photosynthesis-related proteins in the trichome-depleted samples, which can be simply explained by the relatively lower number of chloroplast-containing cells in trichome-enriched samples.

A large number of the known enzymes on the biosynthetic pathway to artemisinin has been detected by combining the information from the two UniProtKB database searches (see Table 2 and Additional file 4) – using the taxonomy *viridiplantae* and *A. annua*, respectively (see Fig. 3). The biosynthesis of artemisinin, as it is currently best understood, is summarized in three phases as shown in Fig. 3, with the enzymes that catalyze each step indicated above each arrow in black. Enzymes appearing in red below each arrow were identified in the *A. annua* taxonomy search (see also Table 2) and enzymes appearing in blue were identified in the *viridiplantae* search, and for the latter, if needed, by homology searching using BLASTp with ‘Artemisia’ as organism (E value < 10^−47^; e.g. searching UniProt # Q84UU4 (α-humulene/(−)-(*E*)-ß-caryophyllene synthase), UniProt # P93665 ((+)-δ-cadinene synthase), UniProt # Q2EPZ0 (cytochrome P450), UniProt # Q42799 (cytochrome P450) and UniProt # Q9ZPB7 (aldehyde dehydrogenase)).

In phase 1 of the biosynthesis of artemisinin, HMGR [18] catalyzes the first transformation in the mevalonate pathway, which is irreversible under physiological conditions, and thereby constitutes a key committed step in the biosynthesis of sesquiterpenes (as well as triterpenes) in the cytosol of higher plants. ADS [19] at the end of phase 1/beginning of phase 2 again catalyzes a committed step which channels the metabolic flux towards the amorphane sesquiterpenes (artemisinin is one such *seco*-amorphane) and away from triterpenes and alternative cyclic sesquiterpene skeletons that are common in *A. annua* (*e.g.* humulanes/caryophyllanes and cadinanes).

It has been known for several years now that the series of three sequential oxidations which converts amorpha-4,11-diene to artemisinic acid (*via* the intermediates artemisinic alcohol and artemisinic aldehyde) in phase 2 of the biosynthesis, is catalyzed by a single cytochrome P450, designated CYP71AV1 [20]. The cytochrome P450s identified from this study, as accession numbers Q42799 and Q2EPZ0, may represent this same enzyme. More recently, it has become clear that dihydroartemisinic acid [21], not artemisinic acid [22], is the true precursor to artemisinin at the start of phase 3 of the biosynthesis. It has been proposed that DBR2 converts artemisinic aldehyde to the alternative product, dihydroartemisinic aldehyde [23, 24]. The 2-alkenal reductase (COLNV1) identified in this study should catalyse this same reaction, and may be involved at this step or was simply identified due to its close homology to DBR2. An aldehyde dehydrogenase such as Q9ZPB7 is then required to convert dihydroartemisinic aldehyde to dihydroartemisinic acid [24, 25].

There is still considerable uncertainty as to the identities of the intermediates involved in the third phase of the biosynthesis of artemisinin, as well as the enzymatic or non-enzymatic nature of these transformations. What is known is that dihydroartemisinic acid can be converted non-enzymatically to artemisinin via an initial oxygenation to the corresponding tertiary allylic hydroperoxide, followed by Hock cleavage, and a second oxidative reaction on the resultant enolic intermediate [26]. However, it is still not clear whether a similar series of spontaneous oxidations occurs *in planta*, or whether enzymes are present to catalyze some steps in this pathway (or whether an alternative series of oxidations occurs *in vivo*). In this regard, it is very interesting indeed to note the high trichome-specific expression of peroxidase 1, which must be a strong candidate as catalyst for the first (and possibly the second) oxidation reaction which is proposed in Fig. 3, if an enzyme were to be involved.

## Conclusions

Using the example of *A. annua*, we have provided further evidence that the choice of sequence database is crucial for successful proteomic analysis. Compared to previously published proteomic data for *A. annua*, we have now shown for the example of a medicinal plant that the employment of an organism-specific database that has undergone extensive curation, leading to longer EST sequences, can greatly increase the number of true positive protein identifications, while reducing the number of false positives.

Most importantly, the presented results substantially increase the (trichome-specific) proteome data available for *A. annua*. An order-of-magnitude more proteins have been identified for trichome-enriched samples, including proteins which are known to be involved in the biosynthesis of artemisinin, as well as other highly abundant proteins, which suggest additional enzymatic processes within the trichomes that are important for the biosynthesis of artemisinin. In particular, the high trichome-specific expression of peroxidases suggests strong enzymatic oxidation activity in trichomes, potentially allowing for effective oxidative reactions in the final phase of the biosynthesis of artemisinin, which have so far been thought to be non-enzymatic in nature.

## Methods

### Solvents and solutions

All solvents used were of HPLC-grade and purchased from Sigma-Aldrich, Poole, UK, except water, which was acquired through Fisher Scientific, Loughborough, UK. The isolation buffer consisted of 200 mM sorbitol (Fluka Biochemika, Buchs, Switzerland), 2 mM sucrose (Sigma-Aldrich), 5 mM succinic acid (Sigma-Aldrich), 5 mM dithiothreitol (Sigma-Aldrich), 1 mM ethylene glycol-bis(2-aminoethyl ether)-*N,N,N’,N’*-tetraacetic acid (Sigma-Aldrich), 0.5 mM Na_2_HPO_4_ (Sigma-Aldrich), 0.1 mM Na_4_P_2_O_7_ (Sigma-Aldrich), 25 mM HEPES (Sigma-Aldrich) and 5 mM MgCl_2_ (Sigma-Aldrich) in water. The precipitation solution was made up with 10 % (w/v) trichloroacetic acid (Fiedel-de Haen, Buchs, Switzerland) and 0.07 % (w/v) 2-mercaptoethanol (Sigma-Aldrich) in cold acetone (Sigma-Aldrich) while the rinsing solution incorporated just 0.07 % (w/v) 2-mercaptoethanol in cold acetone. The solubilization solution consisted of 7 M urea (Sigma-Aldrich) and 2 M thiourea (Sigma-Aldrich) in water.

### Plant material

Branches of the *A. annua* field cultivar Artemis (seed source: Mediplant, Switzerland) were harvested and pooled, and leaves were taken and frozen at −80 °C within 30 min of harvest.

### Isolation of glandular trichomes

A volume of 200 mL of isolation buffer was placed in a 500-mL Schott bottle with 200 μL of protease inhibitor (Calbiochem, Nottingham, UK) and left to stand on ice for 1 hour. After this time, 20 g of frozen *A. annua* leaves were placed into the buffer together with 20 g of glass beads (0.5 mm diameter) (Thistle Scientific, Glasgow, Scotland). The bottle was shaken for 5 min before passing the contents consecutively through 1-mm, 250-μm, 106-μm and 45-μm molecular sieves (Endecotts, London, UK). The liquid was forced through the 106-μm and 45-μm sieves under pressure provided by nitrogen gas. All plant material was returned to the Schott bottle with fresh 200-mL portions of isolation buffer and fresh beads and the process repeated twice. The resulting filtrate was separated into 50-mL tubes and centrifuged for 20 min (2500 *g*, 4 °C). The supernatant was disposed of and the pellet transferred to four 1.5-mL microcentrifuge tubes, which were centrifuged for a further 20 min (2500 *g*, 4 °C). The supernatant was again discarded, leaving the pellets which constituted the enriched glandular trichome sample, each weighing approximately 0.2 g. Leaf material retained by the initial 1-mm sieve was also kept and used as the glandular trichome-depleted sample.

### Environmental scanning electron microscopy

Plant material collected from the 1-mm steel sieve was collected, dried and analyzed by ESEM on a Quanta 600 F instrument (FEI, Hillsboro, OR, USA). For comparison, material obtained from the enriched glandular trichome isolate was also dried and analyzed by ESEM.

### Protein extraction

Each glandular trichome-enriched pellet was crushed using a micro pestle. Aliquots of 2 g of frozen whole leaves (*i.e.* leaves which had not been subjected to the trichome isolation procedure) and 2 g of glandular trichome-depleted sample (prepared as above) were separately flash frozen in liquid nitrogen and ground with a pestle and mortar to obtain a fine powder. A volume of 1.5 mL of precipitation solution was then added to the trichome-enriched samples while 18 mL of precipitation solution was added to the whole leaf and the trichome-depleted samples, respectively. All samples were thoroughly vortexed and then stored at −20 °C for one hour. The trichome-enriched samples were centrifuged for 10 min at 10000 *g* (4 °C) while the whole leaf and trichome-depleted samples were centrifuged for 10 min at 4000 *g* (4 °C). The supernatants from all samples were discarded and the pellets from the glandular trichome-enriched sample were dissolved in 1.5 mL of rinsing solution while the pellets from the whole leaf and glandular trichome-depleted samples were dissolved in 18 mL of rinsing solution. All samples were stored at −20 °C for one hour and then centrifuged for 10 minutes (trichome-enriched samples at 10000 *g*, 4 °C, and whole leaf and trichome-depleted samples at 4000 *g*, 4 °C) before the supernatant was discarded. The previous steps including addition of rinsing solution, centrifugation and discarding of the supernatant were repeated twice for all samples and the resulting pellets were retained from each procedure. The trichome-enriched pellets were then dried under vacuum for 30 min. A volume of 200 μL of solubilization solution was added to each trichome-enriched pellet. Then the samples were vortexed and centrifuged for 10 minutes (10000 *g* at 25 °C) and the supernatants kept. The trichome-depleted and whole leaf pellets were left on the bench for 1 hour for the pellets to dry and 3 mL of solubilization solution was added before the samples were vortexed and centrifuged for 10 minutes (4000 *g* at 25 °C) and the supernatants retained.

The amount of protein in each sample was determined using a Bradford assay.

### Protein digestion

A volume of 210 μL of trichome-enriched material, 227 μL of trichome-depleted material and 139 μL of whole leaf protein extracts with approximately 150 μg of protein each were digested separately. An appropriate volume of a 100-mM dithiothreitol (Sigma-Aldrich) solution was added to each extract in order to obtain a final concentration of 10 mM of dithiothreitol. Each extract was then vortexed and stored at 45 °C for 45 min. The extracts were subsequently left for 5 minutes at room temperature to cool before adding appropriate volumes of a 90-mM solution of iodoacetamide (Sigma-Aldrich) in order to obtain a final concentration of 30 mM of iodoacetamide. A 50-mM ammonium bicarbonate (Sigma-Aldrich) solution was also added to each extract in order to dilute the concentration of urea to approximately 2 M. All extracts were vortexed and left in the dark for a further 45 min. The pH of each extract was checked using pH strips to confirm that the pH remained within the range of 7.5-8. A sequence-grade trypsin (Promega, Southampton, UK) stock solution (200 ng/μL) was added to each extract to obtain a protein-to-trypsin ratio of 100:1 and all extracts were vortexed and left overnight at 37 °C. The digestion of each extract was stopped by adding 10 μL of 0.1 % trifluoroacetic acid (TFA; Sigma-Aldrich).

### LC-MS/MS

Each digested sample was diluted by a factor of 3 using 0.1 % TFA. An aliquot of 1 μL of each sample was injected onto an UHPLC-MS/MS system, consisting of a Dionex 3000RSLC UHPLC system (Thermo Scientific, Hemel Hempstead, UK) and an LTQ Orbitrap XL mass spectrometer (Thermo Scientific) as described previously [27]. Samples were injected in triplicate.

The UHPLC system included an Acclaim PepMap100 C18 100 μm × 2 cm trap column (Dionex, Thermo Scientific) and an Acclaim PepMap C18 75 μm × 25 cm analytical column (Dionex, Thermo Scientific) which was kept at 40 °C. The samples were separated with a flow rate of 0.3 μL/min under a linear gradient elution using 0.1 % formic acid as solution A and 80 % acetonitrile/0.1 % formic acid as solution B for the mobile phase as follows: 4 % B at 0–4 min, 7 % B at 5 min, 50 % B at 120 min, 90 % B at 150–160 min, 4 % B at 160–200 min.

MS/MS analysis was performed on the LTQ Orbitrap XL using an AGC (automatic gain control) target value of 500,000 over 500 ms for the orbitrap and a value of 10,000 over 200 ms for the ion trap. MS spectra (m/z 400–2000 scans) were acquired on the orbitrap mass analyzer set at a resolution of 60,000. The five most intense ions per MS scan with a charge state of ≥2 were sequentially isolated in order of their signal intensity (highest intensity first with a signal intensity threshold set to 5,000 and an isolation window of m/z 3) and fragmented in the linear ion trap by collision-induced dissociation (CID) with a normalized collision energy of 35 %, an activation q value of 0.25 and an activation time of 30 ms. The fragment ions were recorded over the m/z range of 100–2,000. Dynamic exclusion was enabled to minimize redundant sequencing. MS peaks that occurred more than once within 30 s were excluded from selection for fragmentation for 60 s (with an exclusion list restriction to 500 entries).

### Data analysis

All MS/MS spectra were processed using Mascot Distiller software (Version 2.3.2; Matrix Science, London, UK) to convert the raw LC-MS/MS data of each technical replicate for each sample type (trichome-enriched, trichome-depleted and whole leaf sample) into Mascot Generic Files (.mgf files). Searches were then performed against sequence databases using Mascot Daemon (Matrix Science), which combined the database search results from all three technical replicates of each sample type. Mascot (server version 4.2) searches were performed against the UniprotKB database (downloaded on 24. April 2012; 535,698 sequences, 190,107,059 residues), the NCBInr database (downloaded on 07. June 2012), the York *A. annua* (Artemis) contig, the recently published trichome Trinity contig database and an in-house contaminants databases. The York *A. annua* (Artemis) contig database used for this study was established as part of the transcriptome shotgun assembly project from the University of York [13] and was downloaded from the NCBI website http://www.ncbi.nlm.nih.gov/bioproject/39657 (January 2012) and consists of 116,303 RNA sequences from the cultivar Artemis. The recently published trichome Trinity contig database was obtained from Soetaert et al. [14]. Contaminants database searches were performed in order to assess the sample contamination levels due to proteins such as keratins and common protein standards frequently used in the laboratory. Searches were performed using the following parameters: peptide mass tolerance, 10 ppm; MS/MS tolerance, 0.8 Da; peptide charge, +2, +3, +4; missed cleavages, 2; fixed modification, carbamidomethyl (C); variable modification, Oxidation (M); and enzyme, trypsin. Taxonomy of v*iridiplantae* was specified when searching against the *A. annua* contig and UniprotKB databases.

The merged database search results from the trichome-enriched sample were also compared against the merged database results from the trichome-depleted sample and the whole leaf sample by using their Mascot-derived emPAI values and calculating the proportional fold differences for each contig/protein by dividing the emPAI values of the trichome-enriched sample with those values of the trichome-depleted and whole leaf sample, respectively. For this, the Mascot search results were first submitted to the built-in Percolator software, filtered by applying an ‘expect cut-off’ of 0.05 and exported as .csv files using Report Builder within Mascot with a filter of at least 2 ‘significant sequences’.

For each comparison two different lists were created: proteins/contigs that were present in both samples in all triplicates and proteins/contigs that were only present in all triplicates of one sample but in none of the other. In the first list proteins/contigs were ranked according to their proportional difference in emPAI value while in the second list they were ranked according to their absolute emPAI value.

Due to the missing annotation in the *A. annua* contig database a BLASTx search with an E-value cut-off of 0.01 was performed to translate the contigs into known protein homologues. For this search non-redundant protein sequences was specified as the database and Artemisia as the organism. This was performed against the highest ranking contigs of the Mascot database search results comparisons detailed above. The functions of proteins resulting from the BLASTx search were verified using Uniprot Protein KnowledgeBase. Identified proteins with associated GO (gene ontology) molecular function terms were classified into the following categories based on the UniProt database (http://www.uniprot.org): ion binding, small molecule binding, oxidoreductase/antioxidant activity, other catalytic activity, hydrolase activity, structural molecule activity, electron carrier activity, isomerase activity, transporter activity, and protein/peptide binding. The above categories are umbrella terms for more specific GO molecular functions and a number of proteins contributed to more than one GO molecular function class, depending on their (multiple) GO annotation.

MS proteomics data have been deposited as Mascot .dat files to the ProteomeXchange Consortium (http://proteomecentral.proteomexchange.org) *via* the PRIDE (Proteomics Identifications Database) partner repository at the European Bioinformatics Institute (http://www.ebi.ac.uk/pride/), PXD000703.

## Availability of supporting data

The data set supporting the results of this article is available in the PRIDE (Proteomics Identifications Database) partner repository at the European Bioinformatics Institute, PXD000703 (http://www.ebi.ac.uk/pride/).

## Additional files

Additional file 1:
**ESEM images of a fresh (before abrasion with glass beads) A. annua leaf (top), and ‘trichome-depleted’ leaf material (middle) and ‘trichome-enriched’ sample material (bottom) after abrasion with glass beads.**
Additional file 2:
**Number of identified contigs from a triplicate nanoHPLC-MS/MS analysis of three types of** ***A. annua*** **sample material.**
Additional file 3:
**MS/MS Ion Search results of the trichome-enriched sample data against the York Artemis contig database.**
Additional file 4:
**MS/MS Ion Search results of the trichome-enriched sample data against the UniProtKB database (taxonomy:** ***viridiplantae*** **).**
Additional file 5:
**MS/MS Ion Search results of the trichome-enriched sample data against the Artemisia trichome Trinity contig database.**

## Abbreviations

AACT: Acetoacetyl-CoA thiolase
ACT: Artemisinin-based combination therapy
ADS: Amorpha-4,11-diene synthase
AGC: Automatic gain control
ALDH1: Aldehyde dehydrogenase I
CID: Collision-induced dissociation
CYP71AV1: Amorpha-4,11-diene 12-hydroxylase
DBR2: Artemisinic aldehyde delta-11(13) reductase
emPAI: Exponentially modified protein abundance index
ESEM: Environmental scanning electron microscopy
FDR: False discovery rate
FPS: Farnesyl pyrophosphate synthase
HMGR: HMG-CoA reductase
HMGS: (*S*)-3-hydroxy-3-methylglutaryl-CoA synthase
MVAK: Mevalonate kinase
MVAPK: Mevalonate-5-phosphate kinase
MPD: Mevalonate-5-pyrophosphate decarboxylase
PRIDE: Proteomics identifications database
TFA: Trifluoroacetic acid
WHO: World Health Organization

## References

[1] Ansong C, Purvine SO, Adkins JN, Lipton MS, Smith RD (2008). Proteogenomics: needs and roles to be filled by proteomics in genome annotation. Brief Funct Genomic Proteomic.

[2] Bindschedler LV, McGuffin LJ, Burgis TA, Spanu PD, Cramer R (2011). Proteogenomics and in silico structural and functional annotation of the barley powdery mildew Blumeria graminis f. sp. hordei. Methods.

[3] WHO: World Malaria Report 2014. 2014.

[4] Fidock DA (2010). Drug discovery: Priming the antimalarial pipeline. Nature.

[5] Van Noorden R (2010). Demand for malaria drug soars. Nature.

[6] Farhi M, Marhevka E, Ben-Ari J, Algamas-Dimantov A, Liang Z, Zeevi V (2011). Generation of the potent anti-malarial drug artemisinin in tobacco. Nat Biotechnol.

[7] Ro DK, Paradise EM, Ouellet M, Fisher KJ, Newman KL, Ndungu JM (2006). Production of the antimalarial drug precursor artemisinic acid in engineered yeast. Nature.

[8] Paddon CJ, Westfall PJ, Pitera DJ, Benjamin K, Fisher K, McPhee D (2013). High-level semi-synthetic production of the potent antimalarial artemisinin. Nature.

[9] Duke MV, Paul RN, Elsohly HN, Sturtz G, Duke SO (1994). Localization of Artemisinin and Artemisitene in Foliar Tissues of Glanded and Glandless Biotypes of Artemisia-Annua L. Int J Plant Sci.

[10] Wu T, Wang YJ, Guo DJ (2012). Investigation of Glandular Trichome Proteins in Artemisia annua L. Using Comparative Proteomics. Plos One.

[11] Lange BM, Turner GW (2013). Terpenoid biosynthesis in trichomes-current status and future opportunities. Plant Biotechnol J.

[12] Duke SO, Paul RN (1993). Development and Fine-Structure of the Glandular Trichomes of Artemisia-Annua L. Int J Plant Sci.

[13] Graham IA, Besser K, Blumer S, Branigan CA, Czechowski T, Elias L (2010). The Genetic Map of Artemisia annua L. Identifies Loci Affecting Yield of the Antimalarial Drug Artemisinin. Science.

[14] Soetaert SS, Van Neste CM, Vandewoestyne ML, Head SR, Goossens A, Van Nieuwerburgh FC (2013). Differential transcriptome analysis of glandular and filamentous trichomes in Artemisia annua. BMC Plant Biol.

[15] Gershenzon J, Mccaskill D, Rajaonarivony JIM, Mihaliak C, Karp F, Croteau R (1992). Isolation of Secretory-Cells from Plant Glandular Trichomes and Their Use in Biosynthetic-Studies of Monoterpenes and Other Gland Products. Anal Biochem.

[16] Teoh KH, Polichuk DR, Reed DW, Nowak G, Covello PS (2006). Artemisia annua L. (Asteraceae) trichome-specific cDNAs reveal CYP71AV1, a cytochrome P450 with a key role in the biosynthesis of the antimalarial sesquiterpene lactone artemisinin. FEBS Lett.

[17] Wang W, Wang Y, Zhang Q, Qi Y, Guo D (2009). Global characterization of Artemisia annua glandular trichome transcriptome using 454 pyrosequencing. BMC Genomics.

[18] Kong JQ, Cheng KD, Wang LN, Zheng XD, Dai JG, Zhu P (2007). [Increase of copy number of HMG-CoA reductase and FPP synthase genes improves the amorpha4,11-diene production in engineered yeast]. Yao Xue Xue Bao.

[19] Chang YJ, Song SH, Park SH, Kim SU (2000). Amorpha-4,11-diene synthase of Artemisia annua: cDNA isolation and bacterial expression of a terpene synthase involved in artemisinin biosynthesis. Arch Biochem Biophys.

[20] Chang MC, Eachus RA, Trieu W, Ro DK, Keasling JD (2007). Engineering Escherichia coli for production of functionalized terpenoids using plant P450s. Nat Chem Biol.

[21] Brown GD, Sy LK (2004). In vivo transformations of dihydroartemisinic acid in Artemisia annua plants. Tetrahedron.

[22] Brown GD, Sy LK (2007). In vivo transformations of artemisinic acid in Artemisia annua plants. Tetrahedron.

[23] Zhang Y, Teoh KH, Reed DW, Maes L, Goossens A, Olson DJ (2008). The molecular cloning of artemisinic aldehyde Delta11(13) reductase and its role in glandular trichome-dependent biosynthesis of artemisinin in Artemisia annua. J Biol Chem.

[24] Yang K, Monafared RS, Wang H, Lundgren A, Brodelius PE (2015). The activity of the artemisinic aldehyde Delta11(13) reductase promoter is important for artemisinin yield in different chemotypes of Artemisia annua L. Plant Mol Biol.

[25] Kong JQ, Yang Y, Wang W, Cheng KD, Zhu P (2013). Artemisinic acid: A promising molecule potentially suitable for the semi-synthesis of artemisinin. RSC Adv.

[26] Sy LK, Brown GD (2002). The mechanism of the spontaneous autoxidation of dihydroartemisinic acid. Tetrahedron.

[27] Bindschedler LV, Cramer R (2011). Fully automated software solution for protein quantitation by global metabolic labeling with stable isotopes. Rapid Commun Mass Spectrom.

