# Supplementary material for: Proteomic analysis of Artemisia annua – towards elucidating the biosynthetic pathways of the antimalarial pro-drug artemisinin
Source: BMC Plant Biol. 2015 Jul 9;15:175. doi: 10.1186/s12870-015-0565-7 (PMC4496932; doi:10.1186/s12870-015-0565-7)
Supplement: Additional file 1: — ESEM images of a fresh (before abrasion with glass beads) A. annua leaf (top), and ‘trichome-depleted’ leaf material (middle) and ‘trichome-enriched’ sample material (bottom) after abrasion with glass beads. [file 12870_2015_565_MOESM1_ESM.pdf]

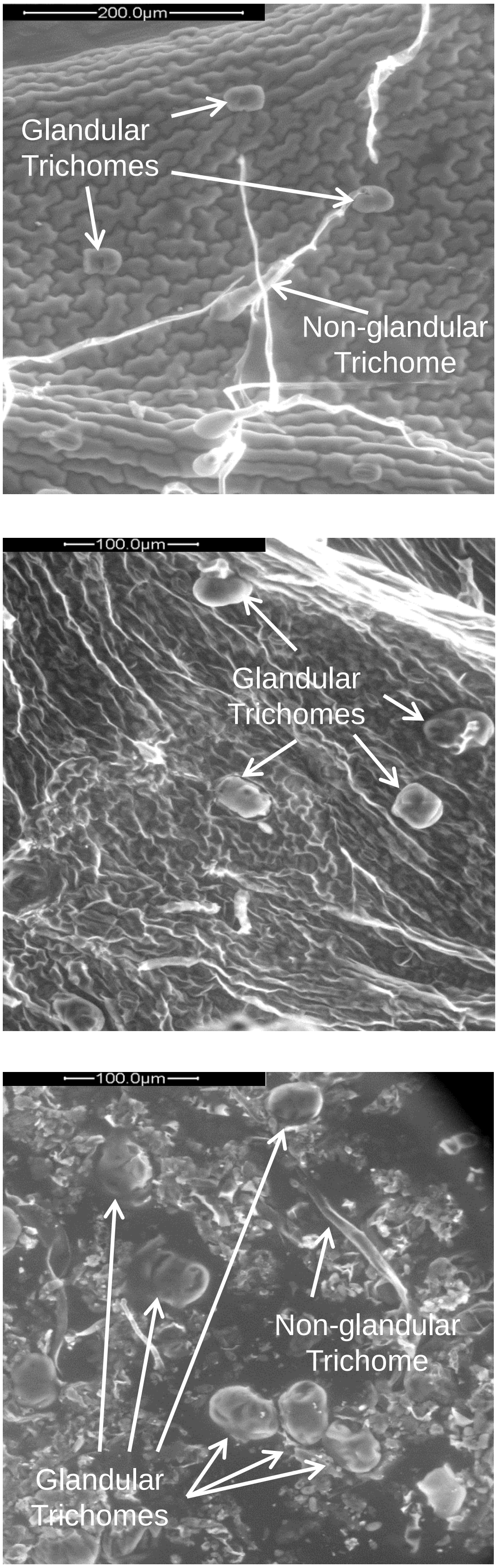

Additional file 1: ESEM images of a fresh (before abrasion with glass beads) A. annua leaf (top), and 'trichome-depleted' leaf material (middle) and 'trichome-enriched' sample material (bottom) after abrasion with glass beads.
