# Supplementary material for: Proteomic analysis of Artemisia annua – towards elucidating the biosynthetic pathways of the antimalarial pro-drug artemisinin
Source: BMC Plant Biol. 2015 Jul 9;15:175. doi: 10.1186/s12870-015-0565-7 (PMC4496932; doi:10.1186/s12870-015-0565-7)
Supplement: Additional file 2: — Number of identified contigs from a triplicate nanoHPLC-MS/MS analysis of three types of A. annua sample material. [file 12870_2015_565_MOESM2_ESM.pdf]

## Additional file 2

Number of identified contigs from a triplicate nanoHPLC-MS/MS analysis of three types of *A. annua* sample material

| | Triplicate 1 | Triplicate 2 | Triplicate 3 | Mean | Standard deviation | Relative standard deviation |
|---|---|---|---|---|---|---|
| **Trichome-enriched samples** | 509 | 513 | 487 | 503.0 | 11.4 | 2.3% |
| **Trichome-depleted samples** | 602 | 631 | 596 | 610.7 | 15.3 | 2.5% |
| **Whole leaf samples** | 486 | 586 | 564 | 545.3 | 42.9 | 7.9% |

For this analysis, Mascot searching was used with an ion score cut-off value of 25. The database used was the Artemis RNA sequence database established through the transcriptome shotgun assembly project from the University of York (downloaded from the NCBI website http://www.ncbi.nlm.nih.gov/bioproject/39657 in January 2012).
